# Development and internal validation of a predictive model of overall and progression-free survival in eribulin-treated patients with breast cancer based on baseline peripheral blood parameters

**DOI:** 10.1007/s12282-025-01678-7

**Published:** 2025-02-20

**Authors:** Keiko Natori, Masataka Igeta, Takashi Morimoto, Masayuki Nagahashi, Sadako Akashi-Tanaka, Takashi Daimon, Yasuo Miyoshi

**Affiliations:** 1 Department of Surgery, Division of Breast and Endocrine Surgery, School of Medicine, Hyogo Medical University, 1-1 Mukogawa-Cho, Nishinomiya, Hyogo 663-8501 Japan; 2 Department of Breast Surgery, Tokyo Women’s Medical University, 8-1 Kawada-Cho, Shinjuku, Tokyo 162-8666 Japan; 3 Department of Biostatistics, School of Medicine, Hyogo Medical University, 1-1 Mukogawa-Cho, Nishinomiya, Hyogo 663-8501 Japan; 4 Department of Breast Surgery, Yao Municipal Hospital, 1-3-1 Ryuge-Cho, Yao, Osaka 581-0069 Japan

**Keywords:** Breast cancer, Peripheral blood parameter, Nomogram, Eribulin, Overall survival

## Abstract

**Background:**

Immune and inflammatory blood parameters have been reported as biomarkers for treatment efficacy. This study aimed to establish a predictive model that includes blood parameters for patients with metastatic breast cancer treated with eribulin.

**Methods:**

A total of 297 patients were enrolled, and their baseline neutrophil-to-lymphocyte ratio, absolute lymphocyte count (ALC), platelet-to-lymphocyte ratio (PLR), prognostic nutritional index (PNI), lymphocyte-to-monocyte ratio (LMR), lactate dehydrogenase (LDH), C-reactive protein (CRP), and clinical data were retrospectively collected.

**Results:**

We constructed nomograms to predict overall survival (OS) and progression-free survival (PFS) using blood parameters, including clinical factors. For OS, menopausal status, hormone receptor status, HER2 status, de novo or recurrent, metastatic site, treatment line, ALC, PLR, PNI, LMR, LDH, and CRP were selected to predict the model. We used menopausal status, hormone receptor status, HER2 status, treatment line, PLR, LMR, LDH, and CRP to predict PFS. Both the OS and PFS of patients according to the risk scores were significantly different (*p* < 0.001). The optimism-corrected C-indices of the nomograms for OS and PFS were 0.680 and 0.622, respectively. The mean time-dependent area under the receiver operating curve values for OS at 1, 2, and 3 years were 0.752, 0.761, and 0.784, respectively, and for PFS at 3, 6, and 12 months were 0.660, 0.661, and 0.650, respectively.

**Conclusion:**

Nomograms incorporating peripheral blood parameters may improve the accuracy of predicting OS and PFS in patients treated with eribulin. Our prediction model may help decision-making for breast cancer patients who are considering eribulin treatment.

## Introduction

Although several agents, including targeted therapies and antibody–drug conjugates, have been established for the treatment of locally advanced or metastatic breast cancer (MBC), chemotherapy still plays an essential role in daily clinical practice. Among several chemotherapies, the determination of effective agents using biomarkers is important for improving patient prognosis. Eribulin, a non-taxane inhibitor of microtubule dynamics, is established as a unique chemotherapeutic agent that achieved longer overall survival (OS) without affecting progression-free survival (PFS) in patients with MBC in the Phase III EMBRACE trial, which compared eribulin with treatment of physician’s choice (TPC) [1]. Because prolonged survival is one of the end points of treatment for patients with MBC, the selection of patients who benefit from eribulin treatment is a critical issue.

Because the immune system, evaluated by tumor-infiltrating lymphocytes, is speculated to contribute to OS in MBC [2], several studies focusing on immune- and inflammation-related parameters in the peripheral blood have been established to be associated with the outcome of patients treated with eribulin. Miyagawa et al. first reported that patients with MBC with a low neutrophil-to-lymphocyte ratio (NLR, < 3) at baseline had significantly better PFS than those with a high NLR (hazard ratio [HR] 0.37, 95% confidence interval [CI] 0.18–0.71, *p* = 0.003) [3]. In addition to NLR, the prognostic significance of absolute lymphocyte count (ALC) was investigated using data from the EMBRACE study, in which OS was compared according to baseline NLR and ALC values in the eribulin and TPC arms [4]. OS was significantly improved in the eribulin arm compared with those in the TPC arm in patients with ALC ≥ 1500/µL (HR 0.586, 95% CI 0.437–0.784, *p* < 0.001), but not in patients with ALC < 1500/µL (HR 1.002, 95% CI 0.800–1.253, *p* = 0.989). A low NLR was significantly associated with prolonged OS in patients treated with eribulin, and similar results were obtained in patients treated with TPC. These observations strongly demonstrated the predictive value of ALC for eribulin efficacy, and that NLR was not a predictive but a prognostic indicator. The significant association between high ALC and improved prognosis was further confirmed in additional reports [5–13].

In addition to the NLR and ALC, the platelet-to-lymphocyte ratio (PLR) and lymphocyte-to-monocyte ratio (LMR) are significantly associated with the prognosis of patients with MBC treated with eribulin [5, 6, 14]. These peripheral blood parameters are derived from blood cell counts calculated using lymphocytes and are established as prognostic factors. Interestingly, the prognostic nutritional index (PNI), which includes the albumin level, was also significantly associated with PFS and OS in patients treated with erbulin [15, 16]. Furthermore, the prognostic significance of an inflammatory marker, C-reactive protein (CRP) [16, 17], and the liver dysfunction marker, albumin–bilirubin (ALBI) score [5] have been reported for eribulin-treated patients.

Many peripheral blood parameters have been reported as predictors of prognosis in patients treated with eribulin. Although a predictive model consisting of multiple parameters seems clinically useful, one has yet to be established. In the present study, we investigated peripheral blood parameters, including ALC, NLR, LMR, PLR, PNI, CRP, and lactate dehydrogenase (LDH) levels, focusing on the predictive significance of prognoses in 297 patients with MBC treated with eribulin. We developed and internally validated nomograms involving peripheral blood parameters to predict the OS and PFS of patients treated with eribulin.

## Patients and methods

### Patient recruitment and eribulin administration

In this pooled retrospective analysis, 297 patients treated with eribulin for MBC were recruited from three institutions (Hyogo Medical University, *n* = 111; Tokyo Women’s Medical University, *n* = 110; and Yao Municipal Hospital, *n* = 76) between July 2011 and October 2022. The inclusion criterion was administration of one or more cycles of eribulin for locally advanced or metastatic breast cancer. Of the 31 human epidermal growth factor receptor 2 (HER2)-positive cancer, 4 patients were treated with eribulin plus anti-HER2 agents (trastuzumab or trastuzumab plus pertuzumab). All patients were diagnosed with invasive breast cancer based on histological findings. Hormone receptor-positive status (i.e., nuclei of more than 1% of cancer cells were positive for estrogen receptor and/or progesterone receptor) and HER2 status were defined based on the ASCO-CAP guidelines [18, 19]. HER2-positive status was defined as an immunohistochemical score of 3+ and HER2-negative status was defined as an immunohistochemical score of 0 or 1+. In situ hybridization was used to definitively determine the HER2 status in conditions where the immunohistochemical score was 2+ ; if the in situ hybridization result was positive, the HER2 status was considered positive. The menopausal status is considered that at the time when breast cancer was diagnosed. Eribulin was administered at a dose of 1.4 mg/m^2^ over 5 min on days 1 and 8 of each 21-day cycle. If intolerable adverse events occurred, the eribulin dose was reduced to 1.1 or 0.7 mg/m^2^ and treatment was delayed in cases with hematological and non-hematological adverse events. The data cutoff date was December 2022.

Eribulin treatment was discontinued owing to progressive disease (*n* = 254), adverse events (*n* = 22), discontinuation at the request of patients (*n* = 8), and other reasons (*n* = 10). Treatment is ongoing in three patients. PFS was defined as the period from the start of eribulin therapy until disease progression or death due to any reason (median, 4.8 months; range = 0.7–38.5 months). We calculated the OS from the start of eribulin treatment to death (median, 15.6 months; range = 1.0–107.4 months).

The Ethics Committee of Hyogo Medical University approved the present study (approval no. 1969) in accordance with the Declaration of Helsinki. The need for written informed consent was waived because clinical data were retrospectively collected.

### Measurements of peripheral blood parameters

In the present study, we collected blood test data on the day before eribulin treatment. Neutrophil, lymphocyte, monocyte, and platelet counts were measured automatically using Sysmex hematology analyzers XN9000, XN-9100, and XE-5000 (Sysmex Corporation, Kobe, Japan) at Hyogo Medical University, Tokyo Women’s Medical University, and Yao Municipal Hospital, respectively. NLR and PLR were obtained by dividing the number of neutrophils (for NLR) or platelets (for PLR) by the number of lymphocytes. The number of lymphocytes was divided by the number of monocytes to obtain the LMR. PNI was calculated by multiplying serum albumin (g/dL) by 10 and adding the ALC (/µL) multiplied by 0.005. Serum albumin, LDH, and CRP levels were measured using LABOSPECT 008, LABOSPECT 008α, and LABOSPECT 006 (Hitachi High-Technologies Corporation, Tokyo, Japan) at the Hyogo Medical University, Tokyo Women’s Medical University, and Yao Municipal Hospital, respectively. The median value (quartile range) of each parameter was 1184 /µL (834–1593) for ALC, 2.81 (1.90–4.09) for NLR, 192 (130–270) for PLR, 3.43 (2.20–4.82) for LMR, 45.0 (40.6–48.4) for PNI, 232 U/L (199 – 311) for LDH, and 0.30 mg/dL (0.10–1.10) for CRP (Table 1).

**Table 1:** Clinicopathological characteristics of patients treated with eribulin according to institutes

	HMU^a^ (*n* = 111)	TWMU^b^ (*n* = 110)	YMH^c^ (*n* = 76)	Total (*n* = 297)	*p* value^d^
Menopausal status					
Pre-	23 (20.7)	45 (40.9)	37 (48.7)	105 (35.4)	0.0003
Post-	86 (77.5)	64 (58.2)	38 (50.0)	188 (63.3)	
Unknown	2 (1.8)	1 (0.9)	1 (1.3)	4 (1.3)	
Hormone receptor^e^					
Positive	84 (75.7)	86 (78.2)	53 (69.7)	223 (75.1)	0.3882
Negative	27 (24.3)	24 (21.8)	22 (28.9)	73 (24.6)	
Unknown	0 (0)	0 (0)	1 (1.3)	1 (0.3)	
De novo or recurrent					
De novo	32 (28.8)	21 (19.1)	20 (26.3)	73 (24.6)	0.2233
Recurrent	79 (71.2)	89 (80.9)	56 (73.7)	224 (75.4)	
Metastatic site					
Non-visceral	26 (23.4)	13 (11.8)	35 (46.1)	74 (24.9)	< 0.0001
Visceral	85 (76.6)	97 (88.2)	41 (53.9)	223 (75.1)	
Number of treatment lines of chemotherapy regimens for advanced or metastatic breast cancer					
1 and 2	75 (67.6)	44 (40.0)	29 (38.2)	148 (49.8)	< 0.0001
3 or more	36 (32.4)	66 (60.0)	47 (61.8)	149 (50.2)	
HER2^f^					
Positive	10 (9.0)	7 (6.4)	14 (18.4)	31 (10.4)	0.0762
Negative	97 (87.4)	101 (91.8)	59 (77.6)	257 (86.5)	
Unknown	4 (3.6)	2 (1.8)	3 (4.0)	9 (3.0)	

^a^Hyogo Medical University

^b^Tokyo Women’s Medical University

^c^Yao Municipal Hospital

^d^*p* values were calculated using Fisher’s exact test

^e^Defined by estrogen receptor and/or progesterone receptor status

^f^human epidermal growth factor receptor 2

### Statistical analysis

Patients’ backgrounds and peripheral blood parameters, including ALC, NLR, PLR, LMR, PNI, LDH, and CRP levels were summarized according to institutes, and their distributions were compared using Fisher’s exact test or the Kruskal–Wallis test. We considered menopausal status (pre- or post-), hormone receptor status (positive or negative), de novo or recurrent status, metastatic site (non-visceral or visceral), number of treatment lines of chemotherapy regimens for advanced or metastatic breast cancer (1 and 2 or 3 and more), HER2 status (positive or negative), and peripheral blood parameters as potential predictors of OS and PFS.

Multivariate imputation using chained equations (MICE) was used to handle the missing data of potential predictors. The number of imputations was set to 100 and the number of iterations within an imputation was set to 20. The imputation model included survival outcome variables and observations of peripheral blood parameters at the end of the follow-up period in addition to the potential predictors listed above, where the peripheral blood parameters were log transformed.

Prognostic factors for OS and PFS were selected as variables with nonzero coefficients in the least absolute and selection operator (LASSO) Cox regression analysis for the stacked dataset, which was created by stacking the imputed dataset. The shrinkage parameter for the LASSO Cox regression analysis for the stacked dataset was obtained by averaging the optimal shrinkage parameters in the LASSO Cox regression analysis for each imputed dataset. In these analyses, a change-point model was applied to the predictor, which indicated a nonlinear association with the outcome in the univariable Cox regression analysis. Metastatic sites (non-visceral and visceral) were excluded from the potential predictors of PFS because the risk of the predictor was not easy to interpret clinically, and the Kaplan–Meier curves for each category of metastatic site indicated that the risk of the non-visceral group was higher than that of the visceral group. These analyses were performed as described in Sect. 23 [20]. Then, nomograms for the prediction of 1-, 2-, and 3-year OS and 3-, 6-, and 12-month PFS were developed based on the predictors determined by LASSO Cox regression models for the stacked dataset.

The overall discriminative capability and internal validity of the nomograms were evaluated using the optimism-corrected concordance index (C-index) values obtained by averaging the optimism-corrected C-index values over all imputed samples. The optimism of an imputed sample was calculated based on 200 bootstrap samples. Calibration plots described the agreement between predicted and observed survival probabilities for OS and PFS at each time point. In addition, prediction accuracy was assessed using the time-dependent area under the receiver operating curve (AUROC) values for OS and PFS. We divided the patients into high-, medium-, and low-risk groups according to the 33.3% and 66.6% quantiles of the risk scorers derived from the nomogram and compared their Kaplan–Meier curves using log-rank tests. Except for the optimism-corrected C-index, these performance evaluations were performed using the first imputed dataset. This simplified method, using a pseudo-complete dataset, was expected to provide a practically acceptable evaluation [20].

The *p* values of less than 0.05 were considered to be statistically significant without considering the multiplicity. All statistical analyses were performed using JMP Pro 16 (SAS Institute Inc., Cary, NC, USA) and R software, version 4.4.0 (www. Rproject.org). The “mice” package was used to perform the MICE. The same packages used in a previous study [21] were used for the LASSO Cox regression analysis, nomogram development, time-dependent AUC calculation, and calibration plot drawing.

## Results

### Clinicopathological characteristics and peripheral blood parameters of breast cancer at baseline

Patient characteristics in each of the three institutes are shown in Table 1. The frequencies of hormone receptor status, de novo or recurrence, and HER2 status were not significantly different. In contrast, the proportions of menopausal status (*p* = 0.0003), metastatic sites (*p* < 0.0001), and number of treatment lines of chemotherapy regimens (*p* < 0.0001) were significantly different among these institutes. The median levels and quartile ranges of each blood parameter according to the institution are shown in Table 2. PLR (*p* = 0.008) and LMR (*p* = 0.007) were significantly different among the institutes, but no significant differences were observed for other parameters, including ALC, NLR, PNI, LDH, and CRP levels.

**Table 2:** Peripheral blood parameters at baseline of patients treated with eribulin according to institutes

		Median (quartile range), number			
	HMU^a^ (*n* = 111)	TWMU^b^ (*n* = 110)	YMH^c^ (*n* = 76)	Total (*n* = 297)	*p* value^k^
ALC level (/µL)^d^	1112 (819–1638), 111	1229 (874–1692), 99	1225 (816–1506), 76	1184 (834–1593), 286	0.675
NLR level^e^	3.06 (2.35–4.42), 111	2.70 (1.66–4.04), 99	2.58 (1.79–3.80), 76	2.81 (1.90–4.09), 286	0.071
PLR level^f^	213 (138–289), 111	197 (137–308), 99	168 (116–221), 76	192 (130–270), 286	0.008
LMR level^g^	3.17 (1.79–4.14), 111	4.03 (2.46–5.42), 99	3.60 (2.36–5.34), 76	3.43 (2.20–4.82), 286	0.007
PNI level^h^	44.6 (40.5–48.0), 111	46.0 (40.5–50.1), 80	45.5 (41.6–50.1), 76	45.0 (40.6–48.4), 267	0.390
LDH level (U/L)^i^	228 (198–294), 82	233 (195–333), 93	238 (205–310), 68	232 (199–311), 243	0.617
CRP level (mg/dL)^j^	0.31 (0.10–1.30), 111	0.30 (0.11–1.15), 61	0.20 (0.06–1.01), 39	0.30 (0.10–1.10), 211	0.686

^a^Hyogo Medical University

^b^Tokyo Women’s Medical University

^c^Yao Municipal Hospital

^d^Absolute lymphocytes count

^e^neutrophil-to-lymphocyte ratio

^f^platelet-to-lymphocyte ratio

^g^lymphocyte-to-monocyte ratio

^h^prognostic nutritional index

^i^lactate dehydrogenase

^j^C-reactive protein

^k^*P* values were calculated using Kruskal–Wallis test

### Identification of predictive factors including clinical and blood parameters

For selecting parameters to incorporate into nomograms, LASSO Cox regression analyses were performed. Menopausal status, hormone receptor status, de novo or recurrence, metastatic site, number of treatment lines of chemotherapy regimen, and HER2 status, ALC, PLR, PNI, LMR, LDH, and CRP levels were identified as factors for constructing nomogram for OS as shown in Table 3. For the PFS nomogram, we incorporated menopausal status, hormone receptor status, number of treatment lines of chemotherapy regimens, and HER2 status, PLR, LMR, LDH, and CRP levels (Table 4).

**Table 3:** Least absolute shrinkage and selection operator (LASSO) Cox regression analyses for predicting overall survival

Variable	Status	Regression coefficient	Hazard ratio
Menopausal status	Pre-, post-	0.302	1.353
Hormone receptor^a^	Positive, negative	0.366	1.443
De novo or Recurrence	IV, rec	0.088	1.092
Metastatic site	Non-visceral, visceral	0.125	1.133
Number of treatment lines of chemotherapy regimens for advanced or metastatic breast cancer	1 + 2, 3 and over	0.367	1.443
HER2^b^	Positive, negative	0.411	1.508
NLR^c^		0	1
ALC^d^ (< 1500) [/µL]		−0.244 × 10^–3^	999.756 × 10^–3^
PLR^e^ (> 200)		0.111 × 10^–2^	100.111 × 10^–2^
PNI^f^ (> 40)		−0.016	0.985
LMR^g^ (< 4)		−0.050	0.952
LDH^h^ [U/L]		0.104 × 10^–2^	100.104 × 10^–2^
CRP^i^ [mg/dL]		0.0530	1.054

^a^Defined by estrogen receptor and/or progesterone receptor status

^b^human epidermal growth factor receptor 2

^c^NLR, neutrophil-to-lymphocyte ratio

^d^ALC, absolute lymphocyte count

^e^PLR, platelet-to-lymphocyte ratio

^f^PNI, prognostic nutritional index

^g^LMR, monocyte-to-lymphocyte ratio

^h^LDH, lactate dehydrogenase

^i^CRP, C-reactive protein

In the column for ‘Variable’, the number in parentheses indicates the change point for the regression coefficient. For example, the regression coefficient for LMR is −0.050 for LMR < 4, and 0 for LMR ≥ 4

**Table 4:** Least absolute shrinkage and selection operator (LASSO) Cox regression analyses of predicting progression-free survival

Variable	Status	Regression coefficient	Hazard ratio
Menopausal status	Pre-, post-	0.231	1.260
Hormone receptor^a^	Positive, negative	0.069	1.071
De novo or Recurrence	IV, rec	0	1
Metastatic site	Non-visceral, visceral	–	–
Number of treatment lines of chemotherapy regimens for advanced or metastatic breast cancer	1+, 2, 3 and over	0.199	1.220
HER2^b^	Positive, negative	0.203	1.226
NLR^c^		0	1
ALC^d^ [/µL]		0	1
PLR^e^		0.439 × 10^–3^	1000.440 × 10^–3^
PNI^f^		0	1
LMR^g^ (< 4)		−0.138	0.871
LDH^h^ [U/L]		0.118 × 10^–2^	100.118 × 10^–2^
CRP^i^ [mg/dL]		0.040	1.041

^a^Defined by estrogen receptor and/or progesterone receptor status

^b^human epidermal growth factor receptor 2

^c^NLR, neutrophil-to-lymphocyte ratio

^d^ALC, absolute lymphocyte count

^e^PLR, platelet-to-lymphocyte ratio

^f^PNI, prognostic nutritional index

^g^LMR, monocyte-to-lymphocyte ratio

^h^LDH, lactate dehydrogenase

^i^CRP, C-reactive protein

In the column for ‘Variable’, the number in parentheses indicates the change point for the regression coefficient. For example, the regression coefficient for LMR is −0.138 for LMR < 4, and 0 for LMR ≥ 4

### Establishment of nomograms for predicting OS and PFS for patients treated with eribulin

**Fig. 1:**
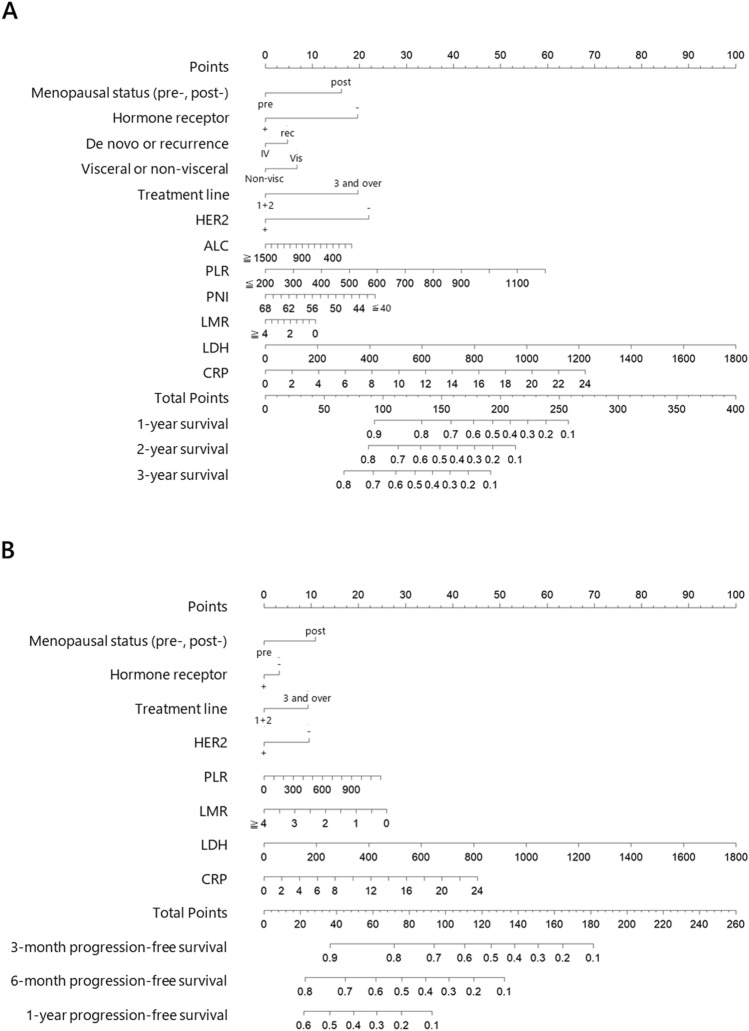
Nomograms to predict overall survival (OS) (**A**) and progression-free survival (PFS) (**B**) in patients treated with eribulin. The nomogram constructed for predicting 1-, 2-, and 3-year OS and predicting values were located at each axis. Point axes were drawn to determine the number of points in each variables. Individual provability was calculated by the total points. Hormone receptor status was defined by estrogen receptor and/or progesterone receptor status

**Fig. 2:**
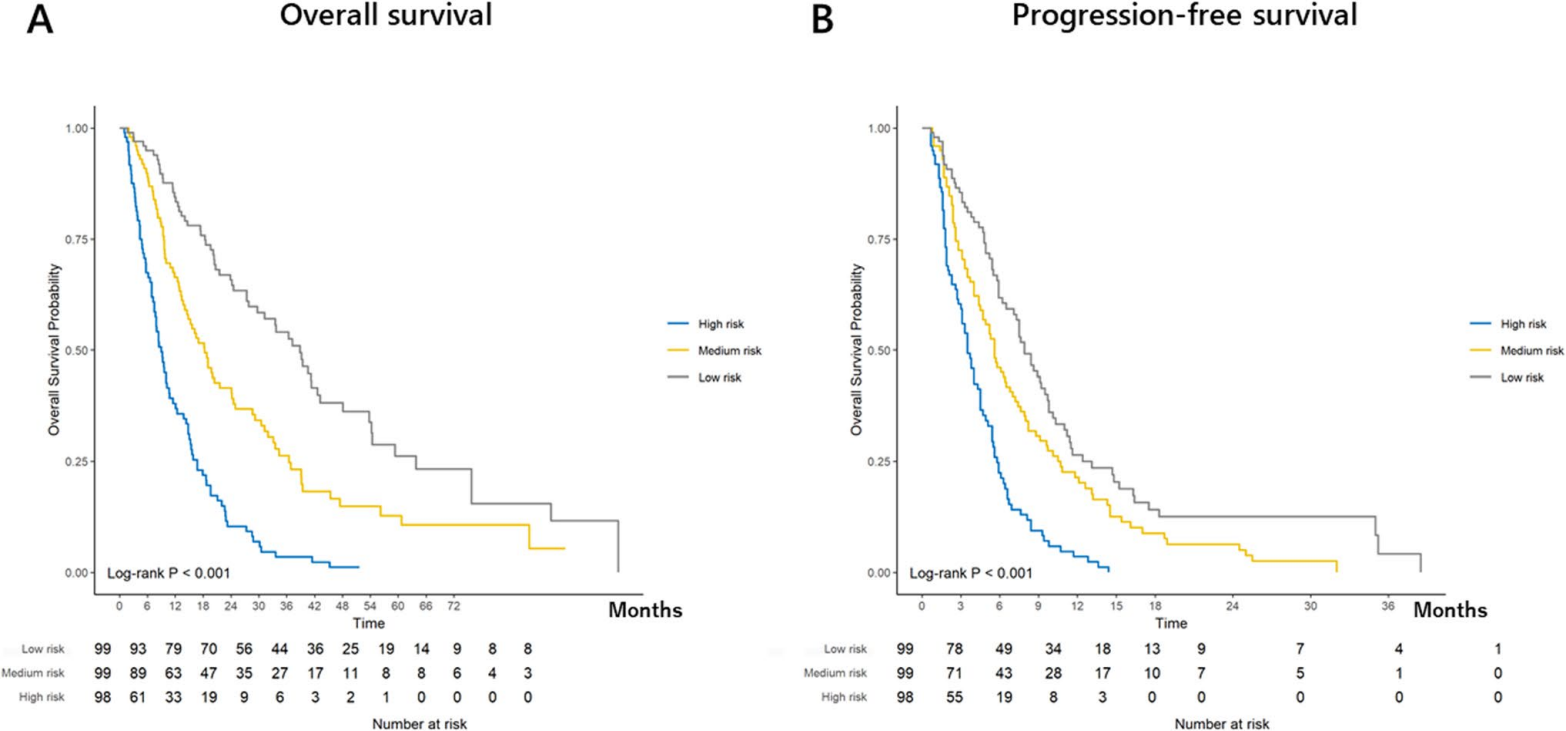
Kaplan–Meier plots of overall survival (OS) (**A**) and progression-free survival (**B**) according to the risks of patients treated with eribulin. Three risk groups were classified based on the total points of nomograms. Significant differences of OS and PFS (*p* < 0.001 each) were obtained depending on the risks

We developed a nomogram for OS using menopausal status, hormone receptor status, de novo or recurrent disease, metastatic site, number of treatment lines of chemotherapy regimens, and HER2 status, ALC, PLR, PNI, LMR, LDH, and CRP levels (Fig. 1A). Based on these factors, this nomogram was designed to predict 1-, 2-, and 3-year survival rates. A weighted number of points was assigned to each factor in the nomogram, and the sum of points was set for the 1-, 2-, and 3-year OS for each patient. Similarly, menopausal status, hormone receptor status, number of treatment lines of chemotherapy regimens, and HER2 status, PLR, LMR, LDH, and CRP levels were used to construct a nomogram predicting PFS (Fig. 1B). Kaplan–Meier plots of OS according to the three groups divided based on risk scorers in the nomogram demonstrated good separation (*p* < 0.001, Fig. 2A). In addition, the accuracy of predicting PFS using the nomogram is demonstrated in Fig. 2B, in which PFS was separated by dividing the risk scores calculated by the nomogram (*p* < 0.001).

**Fig. 3:**
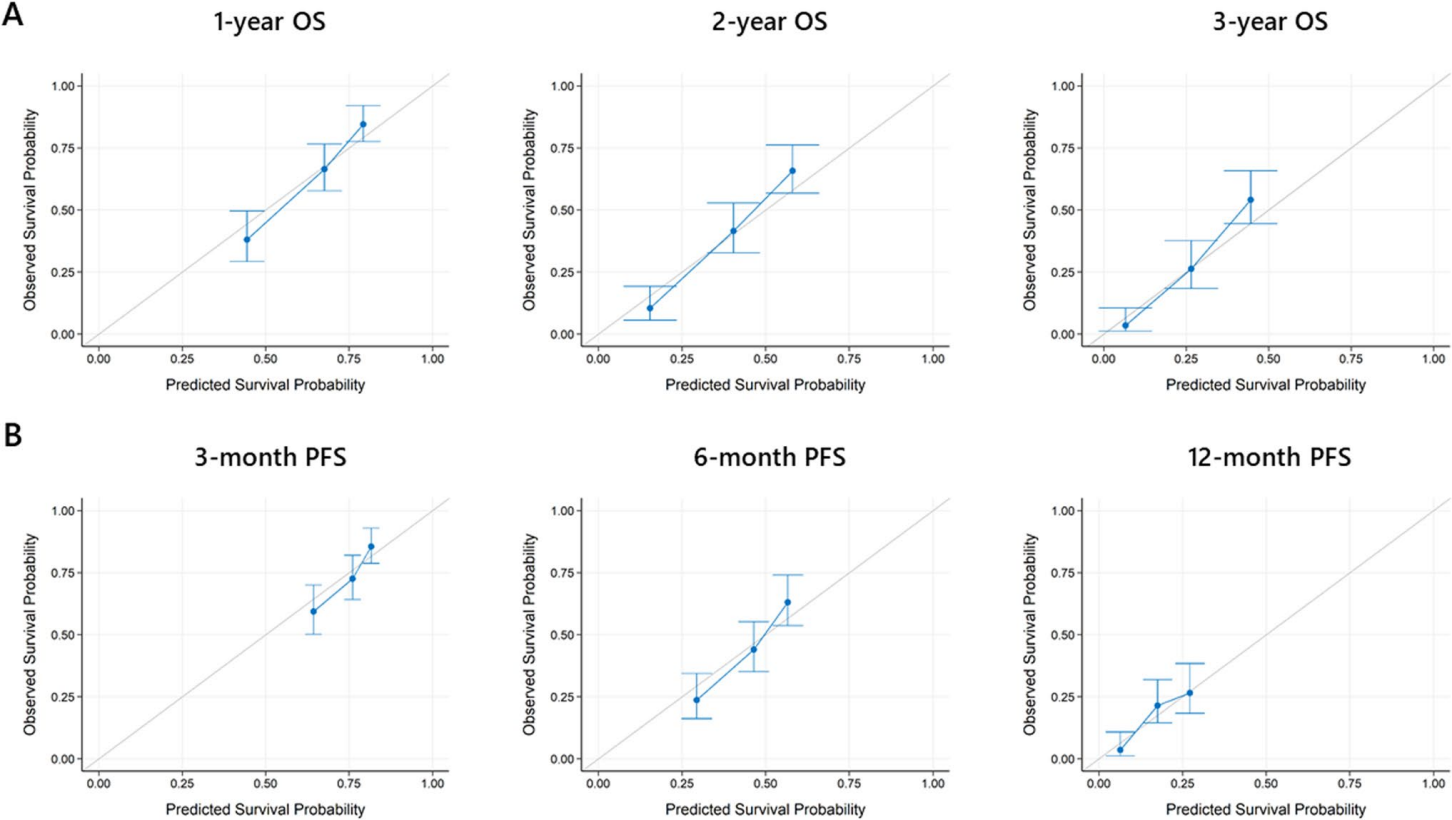
Calibration plots representing predicted and observed prognoses. 1-year, 2-year, and 3-year calibration plots of overall survival (OS) (**A**), and 3-month, 6-month, and 12-month progression-free survival (PFS) (**B**)

**Fig. 4:**
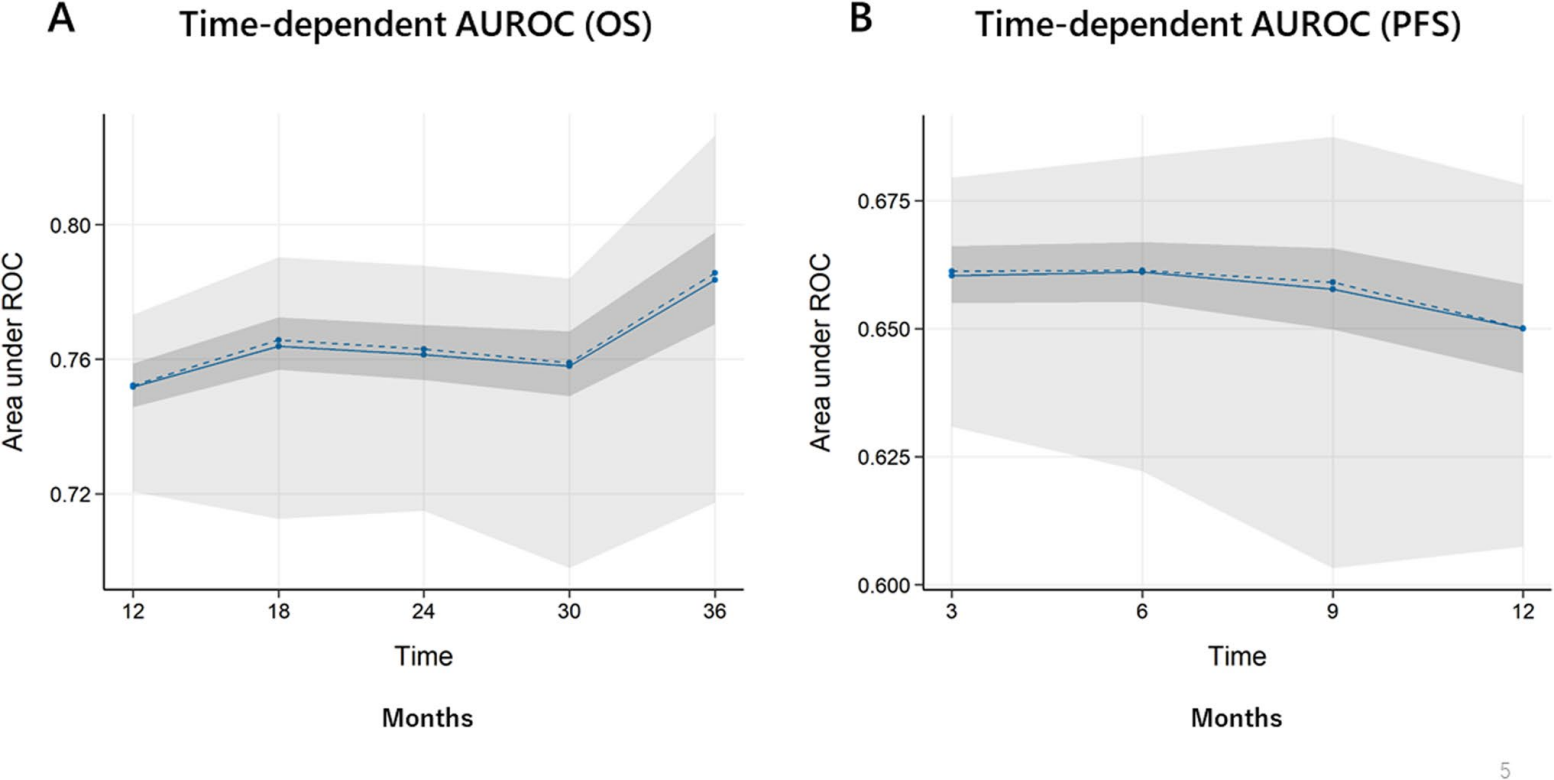
The mean time-dependent area under the receiver operating curve (AUROC) for overall survival (OS) (**A**) and progression-free survival (PFS) (**B**)

The optimism-corrected C-indices of the nomograms for OS and PFS were 0.680 and 0.622, respectively. As shown in Fig. 3A, the calibration plots showed relatively good consistency between the predicted and observed prognoses of 1, 2, and 3 years in the monogram for OS. Similarly, a correlation between the predicted and observed prognoses was observed for PFS at 3, 6, and 12 months (Fig. 3B). The mean time-dependent AUROC values from the bootstrap samples for OS and PFS are shown in Fig. 4A, B, respectively. The time-dependent AUROC values for OS at 1-, 2-, and 3-years were 0.752, 0.761, and 0.784, respectively, and those for PFS at 3, 6, and 12 months were 0.660, 0.661, and 0.650, respectively. Although the C-indices and the time-dependent AUROC values of PFS were lower than those of OS, consistent AUROCs were observed throughout the period.

## Discussion

In the present study, we demonstrated that nomograms constructed using ALC, PLR, PNI, LMR, LDH, and CRP levels, as well as menopausal status, hormone receptor status, de novo or recurrence, visceral metastasis, treatment line, and HER2 status could predict the OS of patients treated with eribulin. Furthermore, PLR, LMR, LDH, and CRP levels, in addition to menopausal status, hormone receptor status, treatment line, and HER2 status were significant predictors of PFS. Although inconclusive, these nomograms may contribute to the selection of patients who benefit from eribulin therapy. ALC has been demonstrated to be a predictive marker of eribulin efficacy in clinical trials [4]. Although it has not yet been confirmed whether these factors are predictive or prognostic markers for other factors, they are useful for predicting the prognosis of each patient, and nomograms that include these blood parameters seem to improve predictive efficacy.

In clinical practice, chemotherapies, including eribulin, are used to treat MBC in the absence of biomarkers. The unique outcome of patients in whom eribulin induced prolonged OS rather than PFS may indicate that an immune reaction against breast cancer plays an essential role, at least in part. Since circulating immune and inflammatory cells in the blood are derived from the microenvironment in breast cancer [22], peripheral blood parameters that reflect the immune status may be suitable as biomarkers for eribulin efficacy. Kaul et al. reported that eriburin-mediated reversal of epithelial-to-mesenchymal transition occurred via suppression of transforming growth factor-β (TGF-β) [23]. The downregulation of TGF-β possibly improved the tumor microenvironment in breast cancer via suppression of cancer-associated fibroblast and endothelial cell function [24], resulting in the stimulation of cancer immunity.

The prognostic significance of immune- and inflammation-related peripheral blood parameters has previously been reported in patients treated with eribulin. As described in the Introduction, ALC, NLR, PLR, LMR, CRP, and PNI levels were candidate predictors of eribulin efficacy. We confirmed that ALC, PLR, and LDH levels were independently associated with PFS using multivariable analysis adjusted for clinical factors (data not shown). Although ALC and PLR were significantly associated with each other, neither ALC nor PLR correlated with LDH (data not shown). We believe that these three parameters are associated with prognosis of patients with eribulin treatment that are mediated through different mechanisms. A high PLR and unfavorable PFS have been reported in HER2-positive advanced breast cancer [25]. Although a high PLR at baseline was significantly associated with poor PFS in patients with metastatic TNBC treated with platinum-based chemotherapy, there was no significant association between PLR and PFS in hormone receptor-positive and HER2-negative control patients [26]. Although not conclusive, PLR may not be a general predictor, but rather a predictor for a subgroup or specific chemotherapeutic agent. Poor PFS has also been reported in patients with high LDH levels during first-line treatment, including endocrine agents and chemotherapies [27], or a change in TNBC [28]. High CRP levels in patients with MBC treated with bevacizumab plus paclitaxel were also associated with poor prognosis [29]. Since the treatment outcomes of immune checkpoint inhibitors differ depending on the levels of peripheral blood parameters, including ALC, PLR, LDH, and CRP [30–34], we speculate that these parameters explain the immune-related mechanisms of action induced by eribulin, at least in part.

The prognostic value of inflammatory markers has been used to develop nomograms for early breast cancer. Cho et al. established a nomogram for early breast cancer to predict disease-specific survival using tumor stage, lymph node metastasis, metastatic stage, progesterone receptor (PgR), and PLR [35]. In this study, among NLR, derived NLR, LMR, and PLR, PLR was the sole independent predictor in the multivariable analysis. Yin et al. established a nomogram predicting disease-free survival was using TNM stage, molecular subtype, and LMR [36]. For MBC, a nomogram predicting OS based on risk factors including albumin and neutrophils has been reported [37]; however, to the best of our knowledge, no other nomogram predicting the prognosis of MBC patients based on peripheral blood parameters has been established to date. PLR, LMR, and NLR differed depending on the institution. The reason for the difference in the values of these parameters is currently unknown. Considering the significant differences in clinical factors, including menopausal status, metastatic sites, and the number of treatment lines of chemotherapy regimens, these factors are speculated to influence peripheral blood parameter values. We demonstrated nomograms including blood parameters, namely ALC, PLR, PNI, LMR, LDH, and CRP levels for OS, and PLR, LMR, LDH, and CRP levels for PFS. These data strongly represent the superiority of prediction accuracy by incorporating blood parameters over clinicopathological factors alone. Shimada et al. investigated blood-based parameters for eribulin efficacy using multivariable analysis and identified albumin-bilirubin grade, hemoglobin level, and PLR, but not CRP, NLR, and ALC as independent predictors [5]. Because PLR, LMR, LDH, and CRP levels were commonly associated with OS and PFS in the present study, we believe that these parameters are strong indicators of eribulin efficacy. In studies involving the development of prediction models using a strategy similar to the one used in this study, the C-indices of the nomograms for predicting OS were approximately 0.6 to 0.7 [37–40], which were comparable to the C-index evaluated in this study (0.680). As this study is the first to develop a prediction model for breast cancer patients treated with eribulin, we believe that the C-index value determined in this study can be used as a target value for prediction models that would be developed in future studies. Although the C-index and the time-dependent AUROC values for PFS were relatively lower than those for OS, consistent AUROCs were observed throughout the study period.

The current study had several limitations. We constructed nomograms with internal validation to predict OS and PFS from pooled data from the three institutes. However, this has not been validated externally using different datasets. In addition, the precise prediction of a combination of blood parameters is unknown. These issues need to be confirmed in future studies that include a larger number of patients using a different dataset.

## Conclusion

We developed and internally validated nomograms to predict the prognosis of patients treated with eribulin. These data may contribute to predicting the prognosis after the start of treatment with eriblin and shared decision-making between patients and physicians when treating MBC with eribulin.

## Data Availability

Data from individual participants were unavailable because the ethics committee did not permit their publication.
